# Physician Awareness and Adherence to Clinical Practice Guidelines in the Diagnosis of Vaginitis Patients: A Retrospective Chart Review

**DOI:** 10.1089/pop.2020.0258

**Published:** 2020-10-16

**Authors:** Paul Nyirjesy, Wendy M. Banker, Tiffany M. Bonus

**Affiliations:** ^1^ Jefferson Vulvovaginal Health Center, Sidney Kimmel Medical College, Thomas Jefferson University, Philadelphia, Pennsylvania, USA.; ^2^ Aurora Research & Consulting, LLC, Spencerport, New York, USA.; ^3^ Horizon Research Insights, LLC, Pittsford, New York, USA.

**Keywords:** vulvovaginitis, bacterial vaginosis, vulvovaginal candidiasis, trichomoniasis, Amsel criteria, NAAT

## Abstract

Vaginitis is one of the main causes of primary care and gynecological visits in the United States. The most common infectious causes are bacterial vaginosis (BV), vulvovaginal candidiasis (VVC), and trichomoniasis. A physician survey was conducted to measure awareness of vaginitis clinical guidelines and availability of in-office point-of-care (POC) diagnostic tools. Participants were asked to perform a chart review to evaluate diagnostic practices for their symptomatic vaginitis patients. A total of 333 physicians and 984 patient charts were included. Physicians were most familiar with VVC and BV diagnostic guidelines; fewer than half were aware of current trichomoniasis guidelines. Although access to POC tools used to evaluate and diagnose vaginitis varied by practice, there was limited access to all 3 tools (microscope, pH test strips, potassium hydroxide solution) required to perform a full Amsel workup for BV (47% obstetricians/gynecologists vs. 32% primary care physicians, *P* < .05). Based on guidelines, 66% of patients evaluated for VVC, 45% of patients evaluated for BV, and 17% evaluated for trichomoniasis received an optimal workup. Among trichomoniasis positive patients, 75% received chlamydia/gonorrhea testing, 42% were tested for HIV, partner therapy was noted in 59% of cases, and 47% returned to be retested within 3 months. Limited awareness of recommended diagnostic practices and lack of access to POC tools contributed to broad guideline nonadherence. This study demonstrates that clinicians commonly fall short of current guidelines and suggests the need for lab-based assessments and appropriate insurance coverage to fill the present diagnostic void.

## Introduction

The most common gynecologic-related diagnosis^1^ in the primary care setting, vaginitis is also one of the most widespread causes for patient visits to obstetrician/gynecologists (OBGYN).^2^ Infectious vaginitis is generally caused by bacterial vaginosis (BV), vulvovaginal candidiasis (VVC), or trichomoniasis.^3,4^ Clinically, vaginitis patients may present with 1 or more troublesome symptoms, including abnormal vaginal discharge, odor, pruritis, discomfort, and pain.^5^ Symptoms can be nonspecific and vary by patient.

Clinical guidelines recommend that BV be diagnosed using Amsel's criteria,^2,6,7^ which are based on presence of 3 or 4 of the following: a homogeneous, thin, white-gray vaginal discharge; a vaginal pH of >4.5; clue cells on saline microscopy; and a positive potassium hydroxide (KOH) whiff test. In research settings, Nugent criteria, which utilize laboratory examination of the Gram stain, are considered the gold standard for diagnosing BV.^2,7,8^ VVC is commonly diagnosed through wet mount (KOH or saline) microscopy or with a positive culture, which is more accurate than wet mount alone.^2,7^ It is recommended that trichomoniasis be diagnosed using nucleic acid amplification testing (NAAT), which is far more sensitive at detecting *T. vaginalis* infections than is saline microscopy.^2,7^

An accurate vaginitis diagnosis can be hindered by several factors within the physician's practice. These include subjective and possibly inaccurate clinician point-of-care (POC) in-office evaluations; diagnosis based only on assessment of patient's symptoms; and a lack of tools and equipment, such as microscopes, to conduct a full patient workup.^9–11^ The presence of mixed infections (2 or more of BV, VVC, and trichomoniasis) or another sexually transmitted infection (STI) can also make accurate diagnosis difficult.^12–14^ Although an appropriate vaginitis diagnosis is essential for clinicians to prescribe the optimal treatment and reduce the likelihood of persistence or recurrence, there is little published information on the extent to which clinicians in nonacademic practices adhere to current guidelines.

The primary objective of this study was to: (1) assess current clinician practices in diagnosing patients with symptoms of vaginitis compared to the practices recommended in clinical guidelines, (2) ascertain clinician awareness of vaginitis clinical guidelines, and (3) assess the availability of diagnostic tools and equipment in the clinician's office.

## Methods

### Study design

In order to address all research objectives, this study included an online survey among physicians followed by online patient chart review forms. The physician survey included questions about their own knowledge and practices related to the evaluation and diagnosis of vaginitis. Physicians then completed the second portion of the survey, a retrospective chart review of symptomatic vaginitis patients. This included in-depth questions about longitudinal events from the patients' initial presentation of symptoms to final diagnosis. The study was piloted among 8 physicians, whose feedback was used to enhance clarity of the survey instrument prior to large-scale data collection. The Western Institutional Review Board determined this study to be exempt from review as the research was survey-based, and physician participants and their patient charts were not identifiable.

### Survey administration

The survey was administered online and completed by a random sample of physicians from M3 Global Research's online panel, which includes more than 4 million health care providers globally. Physicians identified in M3's panel as either an OBGYN or primary care physician (PCP) were sent an email invitation to participate. In order to qualify, physicians had to be in practice for 2 to 35 years, spend at least 70% of time on direct patient care, and have made at least 5 (OBGYN) or 3 (PCP) vaginitis diagnoses in the past month. Physicians were excluded if they were not comfortable pulling prior patient charts from their electronic health record (EHR) system, based on International Classification of Diseases (ICD)-9 or ICD-10 codes, or if they did not have at least 3 vaginitis patients who met study criteria. Physicians who completed the study received $250 for their time.

### Retrospective chart audit

Physicians were instructed to run a report within their EHR that included patients seen at least 12 months prior to survey completion and then chronologically go backward through until they found a patient who was diagnosed with BV, VVC, and/or trichomoniasis. Three patient charts were pulled for study inclusion that met the following criteria: age 18–64; presented with symptomatic vaginitis; received a diagnosis of BV, VVC, and/or trichomoniasis at least 12 months prior to survey completion; and was seen by the reporting physician within the past 12 months (for any reason). Patients diagnosed with a malignancy within 3 years of the vaginitis presentation were excluded.

### Data analysis

All data are unweighted. Results were analyzed using SPSS version 25.0 statistical software (IBM Corp., Armonk, NY); *P* < .05 was considered statistically significant. Analyses were exploratory in nature and reported using descriptive statistics. Subanalyses were performed comparing OBGYN and PCP practices and among patient charts to compare vaginitis evaluation and diagnosis types.

## Results

### Respondent characteristics and demographics

In total, 4031 physicians were invited to this research, 1455 of whom entered the survey (36% response rate). Of these, many did not complete the entire survey (393), did not meet eligibility criteria (517), or entered the survey after the desired number of completed interviews had been achieved (205). The primary reasons for disqualification were physician number of years in practice outside of the study inclusion range (2 to 35) and low comfort level pulling data from historical patient charts using ICD-9/ICD-10 codes. In total, 340 physicians across the United States participated in this research from October 29 to December 3, 2019. Survey responses were reviewed throughout data collection, with 7 physician respondents removed because of poor data quality. A total of 333 physicians (n = 248 OBGYNs and n = 85 PCPs) were included in the final sample.

Each physician personally entered data for 3 patient charts, for a total of 999 patients. Fifteen patients were asymptomatic at the time of presentation, and thus were removed from the study. The final analysis consisted of 984 vaginitis patients. On average, the survey and chart audit instrument took 65 minutes to complete.

### Physician and practice demographics

Table 1 presents physician demographic characteristics. Overall, the physicians were well distributed in terms of practice location, gender, and years in practice. Approximately one quarter were PCP. More than half (56%, n = 185) of the overall group described more than 40 evaluations for vaginitis in a typical month. Sixty-three percent (n = 210) of physicians indicated they work in a private/independent practice and were not affiliated with a hospital.

**Table 1.** Physician Demographic Profile

Characteristic	Total (n = 333)	OBGYN (n = 248)	PCP (n = 85)
Region			
South	30% (101)^a^	29% (73)	33% (28)
Northeast	24% (80)	26% (65)	18% (15)
West	24% (79)	23% (58)	25% (21)
Midwest	22% (73)	21% (52)	25% (21)
Gender			
Male	51% (170)	48% (118)	61%^*^ (52)
Female	49% (163)	52%^*^ (130)	39% (33)
Years in practice			
2–5	14% (48)	15% (37)	13% (11)
6–10	19% (63)	17% (41)	26% (22)
11–15	16% (52)	14% (35)	20% (17)
16–20	20% (67)	22% (54)	15% (13)
21–25	16% (52)	19%^*^ (47)	6% (5)
26–35	15% (51)	14% (34)	20% (17)
Mean (SD)	15.7 (8.4)	16.0 (8.4)	14.9 (8.4)
Vaginitis patient volume ^b^			
1–10	5% (15)	2% (5)	12%^*^ (10)
11–20	12% (40)	10% (25)	18% (15)
21–30	13% (42)	13% (33)	11% (9)
31–40	15% (51)	16% (39)	14% (12)
>40	56% (185)	59%^*^ (146)	46% (39)
Mean (SD)	61.4 (53.3)	63.5 (54.4)	55.2 (49.7)

^a^ Values in parentheses indicate the number of physicians who selected each response choice, unless otherwise noted.

^b^ Number of patients presenting in prior month with suspected bacterial vaginosis, vulvovaginal candidiasis, and/or trichomoniasis.

^*^ Denotes statistical difference between specialties at *P* < .05.

OBGYN, obstetrician/gynecologist; PCP, primary care physician; SD, standard deviation.

Access to POC tools used to evaluate and diagnose vaginitis varied by practice (see Table 2). There was limited access to all 3 in-office tools required to perform a full Amsel workup for a BV diagnosis (47% OBGYN vs. 32% PCP; *P* < .05). Physicians with 2 to 14 years in practice were significantly less likely than those with 15 to 35 years in practice to have access to a microscope (56% vs. 74%; *P* < .05). Compared with OBGYNs, PCPs were significantly more likely to have access to in-office commercial test kits OSOM BVBLUE or OSOM Trichomoniasis rapid test (Sekisui Diagnostics, Burlington, MA), and FemExam pH and amines test card (Cooper Surgical, Shelton, CT).

**Table 2.** Physician Access to Point-of-Care Diagnostics

Diagnostics	Total (n = 333)	OBGYN (n = 248)	PCP (n = 85)
In-office tools			
Potassium hydroxide (KOH)	66% (220)^a^	69%^*^ (172)	56% (48)
Microscope	66% (219)	67% (167)	61% (52)
Vaginal pH test strips	59% (198)	62% (154)	52% (44)
In-office commercial tests			
BD Affirm VPIII Microbial Identification System^b^	20% (65)	21% (51)	16% (14)
OSOM rapid kit (BVBLUE test, Trichomoniasis rapid test)^c^	14% (47)	10% (26)	25%^*^ (21)
FemExam pH and amines test card^d^	10% (33)	8% (19)	16%^*^ (14)

^a^ Values in parentheses indicate the number of physicians who selected each response choice.

^b^ Becton, Dickinson and Company, Franklin Lakes, NJ.

^c^ Sekisui Diagnostics, Burlington, MA.

^d^ Cooper Surgical, Shelton, CT.

^*^ Denotes statistical difference between specialties at *P* < .05.

OBGYN, obstetrician/gynecologist; PCP, primary care physician.

### Physician understanding of guidelines for the diagnosis of vaginitis

**FIG. 1.**
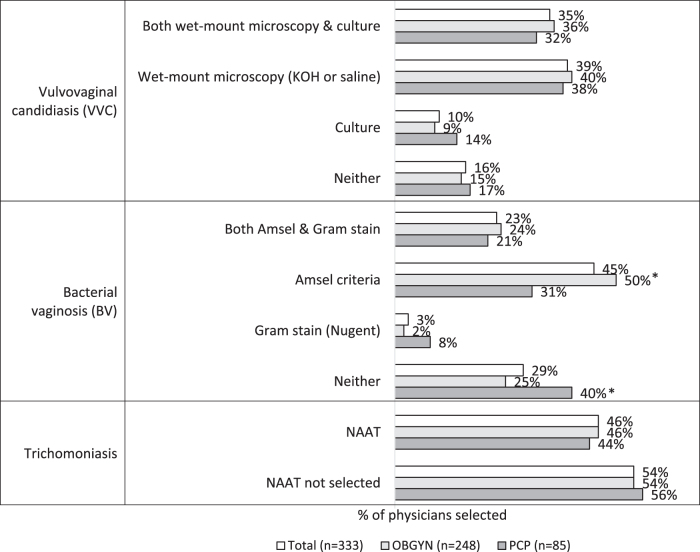
Physician-reported understanding of guidelines for modalities recommended to diagnose each type of vaginitis. Each bar represents the proportion of physicians in total and by specialty who selected the recommended diagnostic studies per vaginitis type. “Neither” or “NAAT not selected” represent the proportion of physicians who did not select a guideline-recommended modality for the given vaginitis etiology. *Denotes statistical difference between specialties at *P* < .05. KOH, potassium hydroxide; NAAT, nucleic acid amplification testing.

Overall, 85% (n = 212) of OBGYNs and 58% (n = 49) of PCPs reported that they follow American College of Obstetricians and Gynecologists (ACOG) guidelines for the evaluation and diagnosis of vaginitis; 60% (n = 51) of PCPs also referred to American Academy of Family Physicians guidelines. Figure 1 illustrates the most common perceptions of recommended diagnostic modalities by diagnosis type, which reflected perceived guidelines during the data collection period (late October to early December 2019). All data for this study were collected prior to the publication of ACOG's 2020 Practice Bulletin.^2^

For VVC, 84% (n = 281) recognized wet mount microscopy (saline or KOH) or culture as the recommended practice (no significant difference by physician specialty). Physicians were less likely to be aware of BV guidelines (84% vs. 71%; *P* < .05). OBGYNs were significantly more likely than PCPs to be aware that Amsel criteria or Gram stain (Nugent criteria) are recommended in BV guidelines (75% vs. 60%; *P* < .05). Physicians were least likely to be aware of trichomoniasis guidelines that recommend NAAT (84% and 71% vs. 45%; *P* < .05); there was no difference by specialty in terms of trichomoniasis diagnostic guideline awareness. More than half (53%, n = 177) of physicians chose saline wet mount as the recommended diagnostic practice for trichomoniasis.

### Patient characteristics

Patient demographics are summarized in Table 3. Eighty-seven percent of patients (n = 853) initially presented to their current physician for vaginitis in 2018, while 8% (n = 81) presented in 2017, and 5% (n = 50) in 2016 or before. Based on physician-obtained history, 19% (n = 191) of patients self-treated their vaginitis using at-home or over-the-counter (OTC) medication and 29% (n = 281) of patients saw at least 1 other health care provider prior to their current physician for symptoms of vaginitis. Few patients (7%, n = 67) had an unintended pregnancy within 6 months prior to their vaginitis presentation.

**Table 3.** Patient Characteristics at Time of Initial Presentation with Vaginitis Symptoms

Characteristic^a^	Total (n = 984)	Final Diagnosis^b^		
		VVC	BV	Trichomoniasis (n = 162)
		(n = 402)	(n = 526)	
Age				
18 to 24	25% (244)^c^	22% (90)	26% (137)	27% (44)
25 to 34	34% (332)	33% (132)	35% (184)	36% (59)
35 to 44	25% (244)	25% (101)	25% (131)	24% (39)
45 to 65	16% (164)	20% (79)	14% (74)	13% (20)
Mean (SD)	33.3 (10.9)	34.3 (11.5)	32.6 (10.3)	31.6 (10.1)
Ethnicity				
White	52% (513)	55% (222)	51% (269)	43% (69)
Black or African American	23% (226)	18% (72)	24% (128)	29% (47)
Hispanic	16% (158)	17% (70)	15% (77)	25% (40)
Other	9% (78)	10% (36)	10% (47)	3% (5)
BMI^d^				
Underweight	1% (9)	1% (6)	<1% (2)	1% (1)
Normal weight	38% (372)	39% (157)	39% (203)	40% (64)
Overweight	25% (248)	23% (93)	26% (139)	21% (34)
Obese	19% (184)	20% (79)	18% (94)	19% (30)
Relationship status				
Single	41% (404)	35% (140)	42% (223)	57% (92)
Married/civil union	32% (314)	39% (155)	30% (156)	19% (30)
Long-term relationship	15% (144)	13% (53)	15% (80)	15% (25)
Divorced or widowed	6% (60)	7% (27)	6% (32)	5% (8)
Birth control usage	63% (616)	63% (255)	62% (325)	63% (102)
Pregnant				
Yes	5% (52)	5% (22)	5% (25)	6% (10)
No	92% (910)	92% (369)	93% (490)	90% (146)
Not sure	3% (22)	3% (11)	2% (11)	4% (6)
Insurance type				
Private	70% (690)	73% (292)	69% (365)	64% (104)
Medicaid	19% (184)	17% (67)	18% (94)	28% (45)
Other	11% (106)	10% (41)	13% (67)	9% (15)

^a^ Based on information available in patient chart from initial presentation visit.

^b^ Coinfection present in 11% of cases, as defined by a final diagnosis of more than 1 vaginitis condition. Given overlap due to coinfection, statistical differences are not assessed between the final diagnosis groups.

^c^ Values in parentheses indicate the proportion of patient charts that fit into each demographic characteristic, unless otherwise noted.

^d^ Unable to assess patient BMI in 17% (n = 171) of patient cases because of missing height and/or weight data.

BMI, body mass index; BV, bacterial vaginosis; SD, standard deviation; VVC, vulvovaginal candidiasis.

At the time of initial vaginitis presentation, 21% of patients (n = 205) reported engaging in high-risk activities within the preceding 3 months, which may or may not have contributed to the onset of vaginitis. “High-risk” activities were based on Centers for Disease Control and Prevention (CDC) definitions of the term: a new sex partner, more than 1 sex partner, a sex partner with concurrent partners, a sex partner who has an STI, or inconsistent condom use.^7^ Patients who were diagnosed with trichomoniasis were significantly more likely to report engaging in high-risk activities, compared with patients who were not diagnosed with trichomoniasis (41% vs. 17%; *P* < .05). Other demographic differences between patients diagnosed with trichomoniasis compared with those who were not include: Black/African American or Hispanic (54% vs. 36%; *P* < .05), single relationship status (57% vs. 38%; *P* < .05), or those who had Medicaid as their primary insurance (28% vs. 17%; *P* < .05).

Physicians ordered STI diagnostic laboratory testing for 39% (n = 385) of patients at the time of initial presentation. STI lab testing included at least 1 of the following: chlamydia, gonorrhea, HIV, syphilis, hepatitis B, human papillomavirus (HPV), herpes simplex virus type 2 (HSV-2), or *Mycoplasma genitalium* (Mgen). Patients were most likely to receive tests for chlamydia (38%, n = 369) and/or gonorrhea (37%, n = 360), followed by HIV (9%, n = 90) and syphilis (9%, n = 86).

### Vaginitis diagnostic practices

**FIG. 2.**
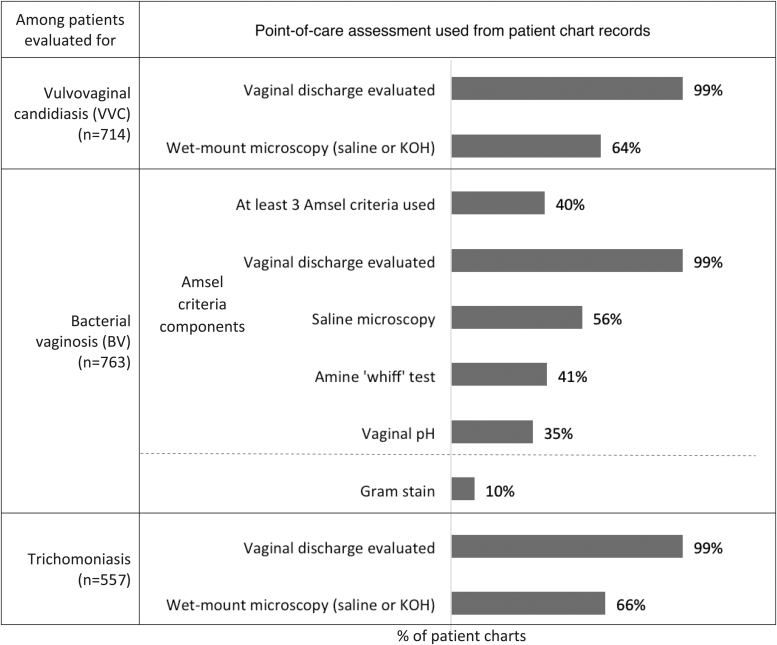
Data collected via patient charts for POC assessment used to make a vaginitis diagnosis based on suspected vaginitis conditions. Each bar represents the proportion of patients in total for each vaginitis etiology evaluated. KOH, potassium hydroxide; POC, point-of-care.

Chart analysis showed that among all patients, 49% (n = 486) were diagnosed based on point-of-care (POC) assessment only, which was defined as at least one of the following: vaginal pH, amine test, saline microscopy, KOH microscopy, Gram stain (in-office), OSOM BVBLUE or OSOM Trichomoniasis rapid test, or FemExam pH and amines test card. In addition to or in lieu of POC assessment, 22% (n = 217) received a BD Affirm test (DNA Probe), 16% (n = 154) received NAAT, and 4% (n = 43) received a vaginitis-related culture or Gram stain ordered from a lab. Nine percent (n = 84) were diagnosed empirically, or based on an assessment of patient-reported symptoms only. See Figure 2 for POC assessments by suspected vaginitis type.

Seventy-three percent (n = 714) of patient cases were evaluated for VVC. Patients considered for VVC had evaluations of vaginal discharge (99%, n = 705) and 64% (n = 459) were assessed with wet mount microscopy (saline or KOH). Yeast culture was used less often (4%, n = 32). BD Affirm was used in 25% (n = 179) and NAAT in 17% (n = 119) of patients. Based on guidelines (saline or KOH microscopy, or yeast culture), 66% (n = 468) of patients received a recommended workup for VVC. OBGYNs were significantly more likely than PCPs to perform a guideline-recommended workup (69% of OBGYN charts vs. 53% of PCP charts; *P* < .05).

Seventy-eight percent (n = 763) of patient cases were evaluated for BV. Among those patients, physicians performed a full Amsel workup (all 4 criteria) in 18% (n = 135) of patients, and 22% (n = 169) had 3 of 4 criteria assessed. Nearly all had vaginal discharge evaluated (99%, n = 754). Gram stain was used in 10% (n = 77). BD Affirm was used in 25% (n = 190) and NAAT in 17% (n = 133) of patients. Based on guidelines (at least 3 of 4 Amsel criteria or Gram stain), 45% (n = 342) of patients received a recommended workup for BV. OBGYNs were significantly more likely than PCPs to perform a guideline-recommended workup (50% of OBGYN patient charts vs. 28% of PCP patient charts; *P* < .05).

Fifty-seven percent (n = 557) of patient cases were evaluated for trichomoniasis. As with other suspected diagnoses, discharge was evaluated in 99% (n = 552) of those considered for trichomoniasis. Saline microscopy was used in 60% (n = 334) of patients considered for trichomoniasis, while BD Affirm was used in 24% (n = 132). Based on guidelines regarding use of NAAT, 17% (n = 95) of patients received a recommended workup for trichomoniasis (no significant difference by physician specialty). Among patients evaluated for trichomoniasis for whom physicians also noted high-risk behaviors within 3 months of presentation, NAAT use remained low (25%, n = 38 patient charts).

Across all patients, those who received NAAT as part of their vaginitis workup were significantly more likely than POC-only assessed patients to be Black/African American (31% vs. 22%; *P* < .05), to have had an unintended pregnancy within the 6 months prior to presenting with vaginitis (12% vs. 4%; *P* < .05), or to have reported high-risk behaviors within 3 months prior to their vaginitis presentation (31% vs. 22%; *P* < .05).

### Vaginitis diagnosis

At the initial vaginitis presentation visit, 93% (n = 919) of patients received a diagnosis of BV, VVC, or trichomoniasis, with a 9% (n = 91) mixed-infection rate (2 or more of BV, VVC, or trichomoniasis). Among patients who received an initial POC diagnosis for which lab testing was ordered, 17% (n = 48) received a change in their vaginitis diagnosis upon return of lab results. Most often, changes in diagnosis were prompted by findings of a different etiology from the initial diagnosis, the addition of a trichomoniasis diagnosis that had not been made prior to lab testing, or an added mixed infection. Ultimately, 53% (n = 526) of patients were diagnosed with BV, 41% (n = 402) with VVC, and 16% (n = 162) with trichomoniasis. The mixed-infection rate was 11% (n = 110). A lower rate of mixed-infection was reported among patients who received POC testing alone and did not receive a molecular-based diagnostic test as part of their workup, compared with those who received a molecular-based test (8% vs. 15%; *P* < .05).

### Trichomoniasis-specific follow-ups

Among patients diagnosed with trichomoniasis, 33% (n = 52) of partners received treatment from a different health care provider. Additionally, physicians provided a prescription refill to 26% (n = 42) of their female patients for partner use. Therefore, partner treatment was definitively noted in 59% (n = 95) of cases. For one quarter (n = 40) of trichomoniasis patients, the physician was unsure if the patient's partner received treatment.

Three quarters of trichomoniasis patients received chlamydia (n = 120) and/or gonorrhea (n = 121) testing, and 42% (n = 68) were tested for HIV (either at initial presentation or upon positive trichomoniasis lab test). Fewer than half (48%, n = 77) returned to be retested for trichomoniasis within 3 months. Thirty-eight percent (n = 62) never returned to be retested for trichomoniasis.

## Discussion

By combining an online survey of physician knowledge of guidelines and a chart review of patients undergoing assessments for vulvovaginal complaints, this study sought to evaluate physician understanding and implementation of current guidelines. This study found that there were certain vaginitis conditions for which physicians were well aware of the guidelines but fell short of executing them and others for which a large percentage of providers were unaware of current recommendations. Differences in practice between OBGYNs and PCPs also were noted in their methods of evaluating vulvovaginitis complaints.

Physicians were most aware of VVC diagnostic guidelines, regardless of specialty. Thus, it was not surprising that patients evaluated for VVC were most likely to receive a guideline-recommended workup of microscopy (saline or KOH) or yeast culture. About one third of patients evaluated for VVC, however, did not receive any guideline-recommended workup, and the test considered the most sensitive for VVC—yeast culture—was seldom (4%) used. As has been emphasized by other investigators, an inaccurate workup or reliance on patient symptoms alone can result in an incorrect or missed VVC diagnosis^10,11,15^ or in the inappropriate use of OTC products.^16^

Knowledge of BV diagnostic guidelines was lower, with OBGYNs more likely to be familiar with the guidelines than PCPs. Although widely available and inexpensive POC tools can accurately diagnose BV in most cases,^17^ many practices continue to lack access to all modalities required for a full Amsel workup, as evidenced in this study and other published literature.^18,19^ The finding that the absence of a microscope is more common among physicians with 2 to 14 years in practice versus those with 15 to 35 years in practice suggests a growing generational shift among providers away from Amsel criteria. More than half of patients (55%) evaluated for BV in this study did not receive an optimal guideline-recommended workup (Amsel or Gram stain). Potential overdiagnosis of BV may lead to improper use of oral or vaginal antibiotics, which may have unpleasant side effects (eg, nausea and dysgeusia) and can, in turn, cause VVC.^20^ A missed or recurrent BV diagnosis can result in lower quality of life.^21–23^

The study team is particularly concerned regarding the lack of guideline awareness and adherence for trichomoniasis—the most common non-viral STI in the United States.^24^ Patients can have minimal symptoms or be asymptomatic,^25–28^ and the sensitivity of a wet mount is approximately 50%–60%.^2,7,29–31^ Saline microscopy has therefore been de-emphasized in older CDC guidelines and is no longer recommended as of 2015.^7^ Perhaps because revised trichomonas guidance is relatively recent, awareness of trichomoniasis evaluation and diagnostic guidelines in this study was significantly lower than for BV and VVC. Not surprisingly, there was very poor adherence to guidelines, with most patients (83%) who were evaluated for trichomoniasis receiving a suboptimal workup. With trichomoniasis in particular, a failure of diagnosis has broad public health implications because an accurate trichomoniasis diagnosis is critical for preventing transmission to new sex partners and alerting existing partners about the need for treatment.^7^

The findings in this study add to the work of other investigators. A recent study conducted by Hillier et al regarding diagnostic testing for vaginal discharge syndromes in practices affiliated with University of Pittsburgh revealed infrequent use of POC tests (15% vaginal pH, 21% KOH whiff test, 17% microscopy) in the evaluation of 303 women. The authors found that 47% of women with a known infectious cause of vaginitis received at least 1 inappropriate prescription and that 34% who did not have an infectious cause incorrectly received either an antifungal or an antibiotic agent.^32^ Somewhat higher, although still inadequate, rates of POC testing were found in the much larger national sample in the present study. Although this study was not intended to assess use of treatments for vaginal infections, the study team feels that the lack of accurate diagnostic testing in this population likely also led to many cases of inappropriate treatment.

In this study cohort, partner therapy was confirmed in only 59% of trichomoniasis-positive cases. Some physicians in this study have adopted Expedited Partner Therapy and provided a refill for the female patient's prescription without first examining her partner. Gaps in guideline adherence also were found related to STI testing and follow-up visits among trichomoniasis positive patients. One quarter of trichomoniasis-positive patients were not tested for chlamydia and gonorrhea, and nearly 6 in 10 (58%) were not tested for HIV, despite guideline recommendations.^7^ With high documented reinfection rates, it is recommended that trichomoniasis patients be rescreened within 3 months after the completion of treatment.^7,26^ This study found that fewer than half (47%) were rescreened within that time frame, and nearly 4 in 10 (38%) never returned to be rescreened.

Ultimately, 17% of the patients in this study study who received a lab test (NAAT, BD Affirm/DNA Probe, Gram stain, or culture) had a change from their initial vaginitis diagnosis. Whether patients who did not receive a lab test may have had their diagnosis changed if they had also received molecular lab testing is unclear. Other published literature reports rates of vaginitis mixed infection (at least 2 of BV, VVC, and trichomoniasis) ranging from 20% to 30%, and an increase in detection of mixed infection with molecular lab testing.^14,33–35^ This study found a relatively low rate of mixed infection (11%) in the total cohort, but also observed that use of molecular lab testing was associated with a higher rate of diagnosed mixed infection. Thus, it can be expected that at least some of the patients who received POC-only workups in the patient cohort had vaginitis diagnoses that were missed and likely would have otherwise been caught with the use of lab-based testing.

### Limitations

Because this study involved a survey approach and a chart review by participating physicians, there were clearly limitations. Despite a large physician sample (n = 333), there may have been selection bias due to physician non-response. Nevertheless, the even distribution of location in the United States and years in practice suggests that a fairly representative sample of physicians was obtained. Interpretation of patient chart results assumes the physician fully entered all information into the chart at the time of visits and also pulled accurate and complete information from their EHR at the time of chart audit completion. Asking physicians to review their own notes within the EHR avoided the limitations of claims database analyses. Study physicians were instructed on how to randomly select patients based on ICD-9 or ICD-10 vaginitis diagnosis codes, in order to mitigate patient-selection bias.

Examining the reliability of physician data entry from chart records, the study team found that physicians sometimes used test-type terminology interchangeably (eg, NAAT/PCR, culture, BD Affirm/DNA Probe and/or vaginitis panel). To aid in the most accurate selection of the lab test evaluation method, the team included example swab and vial images within the online survey instrument. Even with this survey addition, some physicians selected a test type inconsistent with their other responses or within the open-ended description of the patient's workup. In order to improve data accuracy, data cleaning was performed to adjust the lab test type selected based on their summary responses within the survey.

## Conclusion

For at least the past 2 decades, concerns have been raised about the limited use of POC tests.^19^ Past studies have shown that patient self-diagnosis and provider-based telephone diagnosis are also inaccurate most of the time.^16^ Yet during this same time period, providers have received clear and consistent guidance to help correctly diagnose women with vulvovaginal complaints. Study results strongly suggest that those guidelines had little impact on patient care in this cohort. Though this study was not intended to address adverse complications of vaginitis, the study team feels that many women with vulvovaginal complaints may unnecessarily suffer with persistent or recurrent symptoms because of misdiagnoses. Providers should strongly consider turning to other accurate, FDA-cleared diagnostic methods, such as NAAT testing, the approach recommended in the recently published ACOG Practice Bulletin on Vaginitis.^2^
